# PET and SPECT Imaging of Macrophages in the Tumor Stroma: An Update

**DOI:** 10.3390/jcm14145075

**Published:** 2025-07-17

**Authors:** Shaobo Li, Alex Maes, Tijl Vermassen, Justine Maes, Chabi Sathekge, Sylvie Rottey, Christophe Van de Wiele

**Affiliations:** 1 Department of Diagnostic Sciences, Ghent University, Corneel Heymanslaan 10, 9000 Ghent, Belgium; 2 Department of Fundamental and Applied Medical Sciences, Ghent University, 9000 Ghent, Belgium; 3 Department of Nuclear Medicine, AZ Groeninge, 8500 Kortrijk, Belgium; 4 Department of Medical Imaging and Pathology, KU Leuven, 3000 Leuven, Belgium; 5 Department of Medical Oncology, Ghent University Hospital, 9000 Ghent, Belgium; 6 Cancer Research Institute Ghent, 9000 Ghent, Belgium

**Keywords:** tumor stroma, macrophages, PET and SPECT

## Abstract

Tumor-associated macrophages (TAMs) are pivotal immune cells within the tumor stroma, whose dynamic alterations significantly impact tumor progression and therapeutic responses. Conventional methods for TAM detection, such as biopsy, are invasive and incapable of whole-body dynamic monitoring. In contrast, positron emission tomography (PET) and single-photon emission computed tomography (SPECT) offer a non-invasive imaging approach by targeting TAM-specific biomarkers like CD206, TSPO, and CCR2. This review comprehensively summarizes the advancements in TAM-targeted imaging probes, including cell surface markers, metabolic/functional markers, and multifunctional nanoprobe, while assessing their potential in tumor immune surveillance and tumor targeting therapeutic applications. While current probes, including ^68^Ga-NOTA-anti-CD206 and ^64^Cu-Macrin, have exhibited high specificity and theragnostic potential in preclinical and early clinical trials, challenges such as target heterogeneity, off-target effects, and clinical translation persist. Moving forward, the advancement of multi-target probes, optimization of pharmacokinetics, and incorporation of multimodal imaging technologies are anticipated to further enhance the impact of TAM-targeted imaging in precision medicine and tumor immunotherapy, fostering the refinement of personalized treatment strategies and improving patient outcomes.

## 1. Introduction

Tumor-associated macrophages (TAMs) are a major component of the tumor stroma and account for 30–50% of tumor-infiltrating immune cells [1,2]. TAMs can be polarized into two major distinct subtypes: the M1 type (anti-tumor) and pro-tumor M2 type [3]. Stimulation with lipopolysaccharide (LPS) and interferon-gamma (IFN-γ) induces macrophage polarization toward the M1 phenotype [4]. M1 macrophages are typically characterized by high expression of inducible nitric oxide synthase (iNOS) and major histocompatibility complex class II (MHC-II), and they are associated with pro-inflammatory and immune-activating functions. The M2-TAM phenotype is induced by cytokines such as CSF-1, IL-10, and IL-13. M2 macrophages commonly express surface markers such as the macrophage mannose receptor (MMR/CD206), Arginase-1, and folate receptor-β (FRβ), and they represent the predominant pro-tumor phenotype of TAMs [5]. M2-type TAMs contribute to tumor progression through multiple mechanisms, including the suppression of anti-tumor immune responses, the promotion of angiogenesis, and the facilitation of tumor cell invasion and metastasis [6]. Given their important roles in tumor progression and homeostasis, TAMs are considered of major clinical interest for molecular imaging and targeted therapy.

To date, assessment of TAMs in tumor tissue has primarily relied on tissue biopsy and subsequent flow cytometric analysis or immunohistochemistry analysis. However, tissue biopsy is invasive and prone to sample error, and it does not allow whole-body monitoring. These shortcomings can be overcome through both positron emission tomography (PET) and single-photon emission computed tomography (SPECT) utilizing radiolabeled probes designed to bind TAM-specific targets [7]. This review comprehensively summarizes the advancements in TAM-targeted PET and SPECT imaging in the tumor stroma using radiolabeled probes targeting cell surface markers and metabolic/functional markers as well as multifunctional nanoprobes, assessing their potential in tumor immune surveillance and therapeutic applications. A search was performed on PubMed using a combination of the following search terms “macrophage”, “cancer”, “tumor”, “stroma”, “positron emission tomography” and “single photon emission tomography”. Only publications in the field of oncology were included in this review.

## 2. Receptor-Based TAM-Targeted Imaging

An overview of the below-mentioned TAM-related receptors per TAM type is illustrated in Figure S1. An overview of each target in the respective model with the used imaging modalities and agents is given in Table S1.

### 2.1. F4/80 Receptor

The F4/80 is a prototypic member of the EGF-TM7 receptor family, widely recognized as one of the most specific pan-surface markers for identifying murine macrophages [8,9]. Terry et al. developed the novel ^111^ln-anti-F4/80-A3-1 antibody for visualization of tumor-associated macrophages (TAMs) [10]. This radiotracer demonstrated high specificity and affinity with an immunoreactivity of 75% and an IC50 value of 0.58 nM, as evaluated through in vitro binding assays. While it showed promising in vivo biodistribution and micro-SPECT/CT imaging results in an MDA-MB-231 xenograft mouse models, high uptake in the liver and spleen was observed. Although liposomal clodronate pretreatment reduced this uptake and improved the tumor uptake, the liver and spleen signals still affected imaging performance.

### 2.2. CSF1R

CSF1R (colony-stimulating factor 1 receptor) is a protein tyrosine kinase receptor expressed on both M1 and M2 polarized macrophages [11]. By inhibiting the CSF1R axis, the function of TAMs can be downregulated, thereby favorably affecting the immune response within the tumor microenvironment. Waaijer et al. developed the radiolabeled CSF1R monoclonal antibody ^89^Zr-DFO-N-suc-CSF1R-mAb for PET imaging and evaluated its potential for imaging TAMs in a KEP breast cancer mouse model [12]. Their results showed that the radiolabeled antibody accumulated significantly in the spleen, liver, and lymphatic tissues; however, there was lower uptake in the tumor region. Immunohistochemistry and autoradiography analysis revealed that the antibody predominantly concentrated in macrophages and almost completely depleted macrophages within the tumor.

### 2.3. CC2R

The C-C chemokine receptor 2 (CCR2) is a G-protein-coupled receptor (GPCR) that mainly mediates the signaling of chemokines such as CCL2, involved in the chemotaxis and migration of immune cells [13]. CCR2 is expressed in various immune cells, showing a high expression on TAMs of both the M1 and M2 type. Zhang et al. studied CCR2-targeted ^64^Cu-nanoparticles (^64^CuNPs) as positron emission tomography (PET) tracers for gemcitabine-targeted delivery in pancreatic ductal adenocarcinoma (PDAC) KPC and KPCC inoculated mice [14]. The tracer was found to specifically target and distribute in CCR2-positive cells in the tumor stroma, especially on macrophages, significantly enhancing the delivery efficiency of gemcitabine and effectively inhibiting tumor growth.

### 2.4. MMR

The macrophage mannose receptor (MMR, CD206) is a C-type lectin receptor predominantly expressed on M2 polarized macrophages in various tumor models [15]. As a result, CD206/MMR has emerged as a crucial molecular target for TAM-targeted imaging using both SPECT and PET imaging [16].

Movahedi et al. developed a series of nanobody-based molecular probes targeting MMR for in vivo imaging of M2 TAMs using SPECT [17]. The ^99m^Tc-labeled anti-MMR nanobody (Nb cl1) showed the highest binding affinity (KD = 2.31 × 10^−8^ M). It was shown to specifically bind to MMR^+^ TAMs in TS/A and 3LL-R induced tumors in animals, as confirmed by flow cytometry and immunofluorescence co-localization studies. The probe demonstrated strong tumor uptake and specificity in micro-SPECT/CT imaging, with minimal background observed in MMR-deficient mice, validating its receptor-dependent binding. Notably, the uptake was significantly reduced in CCR2^−^/^−^ mice, which lack substantial TAM infiltration, further confirming the TAM origin of the imaging signal. While background uptake in the liver and spleen was observed due to endogenous macrophages, co-injection of unlabeled bivalent nanobodies effectively reduced off-target signals without compromising tumor binding. Zhang et al. developed a NIRF (near-infrared fluorescence)–SPECT dual-modality MMR-targeting monoclonal antibody and assessed its potential for molecular imaging of M2 TAMs in a subcutaneous and lymph node metastasis 4T1 breast cancer model [18]. NIRF imaging and SPECT enabled sensitive detection of the infiltration of M2 polarized macrophages in relapsing tumors and tumor lymph node metastasis. Imaging of M2 macrophages further enabled early prediction of tumor recurrence. Furthermore, initiation of additional radiotherapy, following on M2 macrophages-based response prediction, resulted in a more effective tumor eradication.

Blykers et al. developed the MMR-targeting PET tracer, ^18^F-FB-anti-MMR 3.49 sdAb, by labeling a single-domain antibody (sdAb) with ^18^F-SFB (N-succinimidyl-4-^18^F-fluorobenzoate) [19]. The in vivo biodistribution, tumor-targeting potential, and specificity of this tracer were evaluated in 3LL-R tumor-bearing mice. ^18^F-FB-anti-MMR 3.49 sdAb exhibited rapid renal clearance and significant uptake in MMR-positive tissues and tumors. Moreover, tumor uptake in wild-type mice was markedly higher than in MMR-deficient and CCR2-deficient mice, confirming its specificity for MMR and macrophages. The same group also synthesized ^68^Ga-NOTA-anti-MMR sdAb [20]. In vitro assays with this tracer demonstrated a high binding affinity of approximately 1nM. In vivo studies using wild-type and MMR-deficient mice confirmed the stability and high target specificity, as well as its rapid renal clearance. Based on calculated doses in animals, the extrapolated estimated effective dose in humans was 5.0 mSv for men and 6.3mSv for women for a proposed injected dose of 185 MBq. The authors subsequently performed a phase I clinical trial with ^68^Ga-NOTA-anti-MMR sdAb in seven patients with solid tumors of at least 10 mm diameter. Only one mild headache (Common Terminology Criteria for Adverse Events (CTCAE) grade 1) was reported [21]. The imaging agent exhibited fast blood clearance, allowing high-contrast imaging at 90 min post-injection. The tracer was primarily cleared via the kidneys with minimal hepatobiliary clearance, and the radiation dose (4.2 mSv for men, 5.2 mSv for women) proved comparable to conventional PET tracers. Finally, Parker et al. radiolabeled the MMR targeting peptide RP832c with ^68^Ga for PET imaging using DOTA as chelating agent [22]. In vitro studies demonstrated that ^68^Ga-RP832c remained stable in mouse serum for up to 3 h and exhibited high binding affinity to MMR/CD206, with significant blocking being observed in the presence of excess unlabeled RP832c. PET imaging and biodistribution studies in 4T1 and CT26 tumor models mice revealed strong tracer uptake in tumors and CD206-expressing organs, with a positive correlation between PET signal intensity and CD206/MMR levels.

### 2.5. Folate Receptor β

FRβ is highly expressed in tumor tissues, primarily by M2-like TAMs but also by M1-type macrophages. Over the past two decades, a wide variety of folic acid radio-conjugates have been developed for both SPECT and PET imaging of FRβ [23]. However, recent studies have shown that FRβ can also be expressed in some tumor cells, complicating the differentiation between TAM and tumor cell signals in targeted imaging, thereby affecting signal specificity [24]. This non-exclusive expression pattern may limit the specificity of FRβ-targeted imaging in macrophage-related diagnostics and necessitates further investigation into alternative TAM-selective biomarkers.

### 2.6. TREM2

Triggering receptor expressed on myeloid cells 2 (TREM2) is a pivotal innate immune receptor belonging to the immunoglobulin superfamily, prominently associated with the immunosuppressive M2-type macrophages [25]. Activation of TREM2 was previously shown to promote M1 to M2 macrophage polarization [26]. Shi et al. developed a TREM2-targeted PET tracer, ^68^Ga-NOTA-COG1410, and validated it in a mouse tumor model [27]. Immunohistochemistry and flow cytometry validated the high expression of TREM2 in tumor regions, particularly in the TAM-rich tumor microenvironment. PET/CT imaging was then used to evaluate the in vivo imaging properties of ^68^Ga-NOTA-COG1410. The results showed that the tracer selectively accumulated in tumor tissue, accurately differentiated between tumor and inflammatory regions, and provided high-contrast imaging.

### 2.7. M2pep

Mingxing Huang et al. developed ^68^Ga-DOTA-M2pep as a molecular probe for targeting TAMs [28]. The M2pep peptide was conjugated with the DOTA chelator and subsequently radiolabeled with ^68^Ga to yield ^68^Ga-DOTA-M2pep with high radiochemical purity (>95%). In vitro assays showed that it exhibited high uptake in M2-polarized macrophages, which could be effectively blocked by an excess of unlabeled M2pep, confirming its binding specificity. In vivo micro-PET imaging and biodistribution studies in a B16F10 murine melanoma model revealed that the probe rapidly accumulated in tumor tissue, with prominent uptake observed as early as 1 h post-injection. Moreover, ^68^Ga-DOTA-M2pep demonstrated rapid clearance from the bloodstream, resulting in a favorable tumor-to-background contrast.

## 3. Other Studied Targets for TAM Imaging

An overview of the below-mentioned TAM-related targets in M1-type and M2-type TAMs is illustrated in Figure S1. An overview of each target in the respective model with the used imaging modalities and agents is given in Table S2.

### 3.1. TSPO

Translocator protein (TSPO), also known as the 18-kDa outer mitochondrial membrane protein, is widely expressed in various cell types [29]. Initially, it gained attention due to its high expression in the central nervous system (CNS), where it is primarily found in microglia, astrocytes, and neurons, playing essential roles in cholesterol transport, mitochondrial function regulation, and inflammatory responses [30]. Recent studies have demonstrated that transcription of TSPO is down-regulated in M1 macrophages but remains unaltered in M2 type macrophages, rendering TSPO a potential target for in vivo visualization of M2-like macrophages in the tumor stroma. Also, TSPO was shown to be present in various human cancer cells, potentially reducing the specificity of TSPO-imaging for M2 macrophage detection. Nevertheless, limited available data suggest that the impact of TSPO expression in some tumor types may not prove problematic for M2 imaging. The TSPO targeting ligand ^18^F-DPA-714 PET was utilized to detect M2-polarized TAMs in a triple-negative breast cancer tumor model (TNBC) with the aim of providing insights into tumor stratification and prognosis evaluation [31]. PET imaging following injection of the ligand demonstrated excellent tumor-to-background contrast and consistent uptake within tumor lesions. To validate the in vivo findings at the cellular level, histological analyses were performed using immunohistochemistry for CD68 (macrophage marker) and CD163 (M2 marker), as well as in vitro autoradiography with ^3^H-DPA-714 and ^3^H-PK11195. These analyses revealed a dominant infiltration of M2-polarized macrophages in most TNBC tissues, supporting the hypothesis that the ^18^F-DPA-714 PET signal reflects the abundance and activity of immunosuppressive TAMs. Lanfranca et al. established a pancreatic ductal adenocarcinoma model using transgenic mice harboring pancreas-specific mutations in KRAS and p53 and derived a murine tumor cell line lacking endogenous TSPO expression [32]. These cells were implanted into syngeneic mice to form tumors, followed by intravenous injection of the TSPO-specific PET-radioligand ^11^C-PBR28. Tumor uptake of the tracer was assessed through autoradiography, ex vivo tissue analysis, and micro-PET imaging. The resected tumors were found to be rich in macrophages, as confirmed by immunohistochemistry and flow cytometry. Immunoblotting revealed that TSPO expression in murine macrophages increased upon activation and polarization. Autoradiography of tumor sections confirmed ^11^C-PBR28 uptake, and whole-mount analysis demonstrated its capability for clear tumor localization. Importantly, repeat experiments in CD11b-deficient mice showed significantly reduced tracer uptake, validating the macrophage specificity of the probe.

### 3.2. Glucose Metabolism

TAMs significantly influence the metabolic characteristics of tumors within the tumor microenvironment, playing an important regulatory role, particularly in glucose metabolism pathways. A limited number of studies have shown a significant positive correlation between the density of TAMs in tumor tissues and the uptake levels of ^18^F-FDG PET in lung adenocarcinoma and SCCHN, suggesting that TAMs at least in these tumor subtypes may promote glycolysis in tumor cells amongst others through the release of inflammatory factors such as TNF-α [33,34]. Additional research has shown that the removal of TAMs from tumors using liposomal clodronate significantly decreases the tumor’s glycolytic activity and hypoxic levels and enhances the tumor’s response to immunotherapy [34]. These results support the close relationship between TAMs and the tumor metabolic microenvironment.

### 3.3. Phagocytosis

M2 polarized macrophages have previously been shown to have significantly higher phagocytic activity compared with M1 macrophages [35,36]. This differential phagocytic ability of M2 as opposed to M1 macrophages has laid the basis for the design and application of nanoparticle-based imaging agents for in vivo M2-TAM imaging.

^89^Zr-PL-HDL and ^89^Zr-AI-HDL are nanoparticle probes based on recombinant high-density lipoproteins (rHDL) designed for TAMs PET imaging through phagocytosis [37]. Medina et al. synthesized two ^89^Zr-labeled rHDL probes for TAM imaging: ^89^Zr-AI-HDL labeled apolipoprotein A-I (apoA-I) with^89^Zr, and ^89^Zr-PL-HDL which incorporated ^89^Zr in phospholipids. Validation of these tracers in an orthotopic model of breast carcinoma showed significant radioactive accumulation in the tumor region within 24 h of injection for both probes, with ^89^Zr-AI-HDL (16.5 ± 2.8%ID/g) exhibiting a higher uptake rate than ^89^Zr-PL-HDL (8.6 ± 1.3%ID/g). Tissue section analysis and flow cytometry confirmed that TAMs took up the rHDL probe significantly more than tumor cells (approximately 6.8 times higher), contributing 40.7% (^89^Zr-AI-HDL) and 39.5% (^89^Zr-PL-HDL) of the total probe uptake in tumor tissues. These results indicate that the ^89^Zr-rHDL probe specifically targets TAMs, offering a precise, non-invasive imaging tool for breast cancer TAMs PET imaging.

Kim et al. aimed at targeting TAMs using ^64^Cu-Macrin, a nanoparticle-based probe optimized pharmacokinetically to enhance its specific uptake by macrophages [38]. The authors combined PET imaging, high-resolution confocal microscopy, and tissue-clearing techniques in a mouse lung cancer model, finding that ^64^Cu-Macrin’s selective uptake by macrophages exceeded 90%, with uptake correlated strongly with macrophage content in tissues (R^2^ > 0.9). Additionally, ^64^Cu-Macrin effectively monitored dynamic changes in TAMs after chemotherapy and γ-irradiation treatment, with TAM numbers increasing by 180% to 650% post-treatment. In nanodrug delivery research, TAM-rich tumors showed more than a 700% increase in nanodrug uptake, including PEGylated liposomal doxorubicin and TAM-targeted TLR7/8 agonist R848 nanoparticle formulations. This suggests ^64^Cu-that Macrin can be used not only for non-invasive TAMs PET imaging but also to predict and optimize TAM-based nano-therapy strategies.

Landon et al. designed MAN-LIPs (mannose-modified liposomes) labeled with ^64^Cu for PET imaging of TAMs [39]. Additionally, fluorescent dyes embedded in the lipid bilayer enabled subsequent fluorescence microscopy analysis. In a murine lung cancer model, in vivo PET imaging and ex vivo organ analysis were performed following the injection of MAN-LIPs. The results demonstrated significant accumulation of MAN-LIPs in TAMs within tumor sites, whereas uptake in normal lung tissue was minimal. These findings indicate that MAN-LIPs exhibit excellent targeting specificity for TAMs in imaging applications while also holding potential as a drug delivery platform.

## 4. Discussion

TAMs are key immunoregulatory cells within the tumor stroma, with their phenotypic distribution closely linked to therapeutic responses and disease prognosis [40]. As such, TAMs have emerged as prominent targets in molecular imaging research.

A limited number of studies have shown significant positive correlations between the density of TAMs in human tumor tissues and their uptake levels of ^18^F-FDG PET in lung adenocarcinoma and SCCHN, respectively [33,34]. M1 macrophages have been previously shown to rely on aerobic glycolysis, whereas in M2 macrophages, the main source of energy is oxidative phosphorylation and fatty acid oxidation [41]. In tumor models, hypoxia was shown to facilitate macrophage migration into the tumor as well as polarization into M2 type macrophages, promoting tumor growth due to the production of a large number of mitogenic, angiogenic, and prometastatic cytokines and enzymes [42]. Thus, depending on the degree of hypoxia, the total number of macrophages as well as the ratio of M1/M2 macrophages may vary significantly. Furthermore, cancer cells continuously adapt to changes in environmental pressures and alterations in growth conditions in order to produce sufficient ATP for their survival and growth. As a result, the ratio between glycolysis and oxidative phosphorylation as well as between glucose/glutamine and fatty acid are continuously changing [43]. Given the total uptake of 18F-FDG by the tumor tissue reflects uptake by both tumor tissue and its environment, including TAMs, and its dependence on various continuously changing factors as described above, it is not specific enough to provide relevant information on macrophage infiltration in the tumor stroma. Potential strategies that may distinguish the in vivo 18F-FDG PET/CT imaging metabolic changes caused by TAMs from those caused by the tumor cells themselves include administration of treatment modalities that polarize TAMs towards an anti-tumorigenic phenotype, e.g., blocking the CD47-SIRPα axis, or that induce depletion of TAMs in the tumor microenvironment, e.g., biphosphonates and trabectedin [44].

Similar to 18F-FDG PET/CT imaging, the fact that TSPO and FRβ are also expressed on various cancer cells to a variable degree may limit their usefulness as a targets for TAM imaging in tumors, although this may not be the case for all tumor types. While the F4/80 receptor is a pan-marker of murine macrophages, its human homologue EGF-like module containing mucine-like hormone receptor (EMR1) is absent on human macrophages and restricted to eosinophilic granulocytes in humans [45]. Other biomarkers that have been studied as a target for TAM-imaging using PET and SPECT that are expressed on both M1 and M2 type macrophages include CSF-1R and CCR2. Ligands targeting these receptors will provide an estimate of the degree of TAM infiltration in the tumor stroma, which is also the case for phagocytosis-based tracers, altough in the latter case, the contribution of M2 TAMs is likely to outweigh that of M1 TAMs. While the high degree of infiltration of macrophages in the tumor stroma has been previously correlated to be a poor patient prognosis [46,47], whether or not information derived from PET and SPECT images using CSF-1R and CCR2 targeting ligands can provide independent additional prognostic information in addition to already well-established prognostic markers remains to be proven. Furthermore, given that CSF1R and CCR2 are also broadly expressed within the mononuclear phagocyte system, their anticipated high physiological uptake in organs such as the liver and spleen may compromise image contrast and specificity [12,14]. It is interesting that while this review paper focuses only on TAM imaging in oncology, the potential of various ligands targeting TAMs, including some of the those mentioned in this review paper, is currently also being assessed in ongoing clinical trials in other fields, e.g., inflammation imaging [48].

Given their association between immune tolerance, angiogenesis and tumor progression, more recent imaging strategies have primarily focused on targeting the M2 type macrophages, with CD206 being the most widely targeted biomarker for probe development. A recent systematic review and meta-analysis performed on various tumor types, despite heterogeneity in the published results, identified worse overall and disease-free survival for patients with increased CD206-expressing TAMs in their tumor micro-environment [49]. While CD206 is a pan-marker of M2 macrophages and has so far proven to be the most successful target for PET imaging in the field of oncology, the growing understanding of macrophage polarizing conditions does not exclude the discovery of novel macrophage phenotypes and accordingly, more suitable targets for imaging [44]. To date, the most promising tracer targeting M2 macrophages is ^68^Ga-NOTA-anti-MMR sdAb, which has been validated in preclinical tumor models, demonstrating high specificity, and has also been safely injected in healthy volunteers. Mannosylated radiolabeled compounds can also bind to other pathogen-recognition receptors such as CD209, while 68Ga-NOTA-anti-MMR-sdAb specifically binds to CD206 with high affinity. In addition, sdAbs have further advantages such as high thermostability, good solubility, and strictly monomeric behavior; their small size of approximately 15 kDa allows a high tumor penetration rate and rapid clearance from the blood, making them ideal tracers for imaging using PET and SPECT. Their potential to target intracellular epitopes as well as epitopes concealed from mAbs in protein structures is related to their long CDR3 loops, relatively low production cost, and ease of genetic engineering. Inversely, disadvantages when considering their use for in vivo imaging in humans include the restriction of the paratope of sdAds to 110 amino acids, limitations to their extent of manipulation and engineering, which often drastically compromise their antigen-binding affinity, and their low propensity to bind small molecules, which is probably related to their dominant convex surface topology. In addition, the fact that sdAds are of non-human origin demands a thorough exploration of their immunogenicity and toxicity for in vivo use [50]. Targeting M2 type macrophages through imaging will not only enable assessment of the tumor immune landscape but will also provide critical insight for predicting therapeutic response. Furthermore, emerging immunoregulatory molecules such as TREM2 may also prove of relevance for M2 type macrophage imaging in oncology [27]. Finally, TAM subtypes were previously shown to vary from one tumor type to another, and it is assumed that TAMs can acquire different functional phenotypes depending on their location in hypoxic or perivascular regions, inside the tertiary lymphoid structures, in the tumor nest, or at the invasive front. Hence, a better understanding is required of their function and spatial interaction with other cells in the tumor micro-environment, e.g., fibroblasts at different sites within the tumor and the molecular pathways. Spatial transcriptomics methods and single-cell RNA sequencing, among other methods, may help to select imaging probes more likely to be of clinical interest in different types of tumors [51].

Importantly, advancements in imaging technology are tightly linked to ongoing optimization of probe design. The molecular size and physicochemical properties of radiotracers directly affect their tissue penetration, target affinity, and in vivo biodistribution. Small-molecule probes such as ^18^F-DPA-714 can diffuse passively across cell membranes and bind to TSPO on the mitochondrial membrane, enabling efficient imaging of activated immune cells [31]. In contrast, nanoprobes based on single-domain antibodies (sdAbs), such as ^64^Cu@CuOx-ECL1i, utilize phagocytosis-mediated uptake by macrophages to enhance TAM signal intensity [14]. On the other hand, conventional antibody-based probes, due to their large molecular size, limited tissue permeability, and slow clearance, typically require long-lived radionuclides such as ^89^Zr for delayed imaging, making them more suitable for chronic disease monitoring or immunotherapy evaluation. The selection of the radionuclide is equally critical, as the physical properties of isotopes determine their compatibility with different probe types. Common positron emitters include ^68^Ga, ^18^F, and ^11^C, whereas ^99m^Tc and ^111^In are widely used in SPECT imaging. Long half-life isotopes such as ^89^Zr and ^125^I are frequently employed to label antibodies for tracking their prolonged biodistribution in vivo. ^11^C (t_1⁄2_ = 20.4 min) is suitable for rapidly diffusing small molecules, while ^89^Zr (t_1⁄2_ = 78.4 h) is more appropriate for long-term tracking of large biomolecules, enabling comprehensive immune cell visualization.

Despite significant progress, the translation of TAM imaging from preclinical studies to clinical applications faces several challenges. Firstly, interspecies differences in macrophage markers, such as F4/80—specific to murine macrophages but lacking a human homolog—limit the clinical development of related antibodies [10]. Secondly, although xenograft models partially mimic the human tumor microenvironment, they fail to fully capture the complexity of the human immune system. To date, only a few TAM-targeted probes, including ^68^Ga-NOTA-anti-MMR-sdAb, ^18^F-DPA-714, ^11^C-PBR28, and ^18^F-FDG, have entered clinical evaluation, and most remain in early-phase trials with small sample sizes, underscoring the urgent need for larger, multicenter studies to validate their safety and efficacy [20,21,31,32]. In the future, AI and machine learning are expected to improve the safety and efficacy assessment of imaging probes, thus accelerating the clinical implementation of these probes [52].

Looking forward, the advancement of TAM-targeted molecular imaging will require coordinated progress across multiple fronts. On one hand, novel targets and probes with improved specificity and reduced off-target uptake are needed. On the other hand, integrating multimodal imaging technologies such as PET/MRI and SPECT/CT, alongside emerging tools like artificial intelligence, will enhance our capacity to quantify the spatiotemporal dynamics of TAMs. Ultimately, the combination of imaging biomarkers with immunotherapeutic strategies, e.g., CSF-1R inhibitors, holds the promise of achieving more precise and individualized cancer management.

## Supplementary Materials

The following supporting information can be downloaded at https://www.mdpi.com/article/10.3390/jcm14145075/s1. Figure S1: Overview of cell surface and intracellular biomarkers used for radionuclide targeting of TAMs; Table S1: Cell surface biomarkers of TAMs; Table S2: Other TAM-related biomarkers.
