# Supplementary material for: PET and SPECT Imaging of Macrophages in the Tumor Stroma: An Update
Source: J Clin Med. 2025 Jul 17;14(14):5075. doi: 10.3390/jcm14145075 (PMC12294810; doi:10.3390/jcm14145075)
Supplement: Supplementary file 1 [file jcm-14-05075-s001.zip › jcm-3707352-supplementary.pdf]

Journal of Clinical Medicine

Supplementary Material

PET and SPECT Imaging of Macrophages in the Tumor Stroma: An Update

1. Supplementary figures

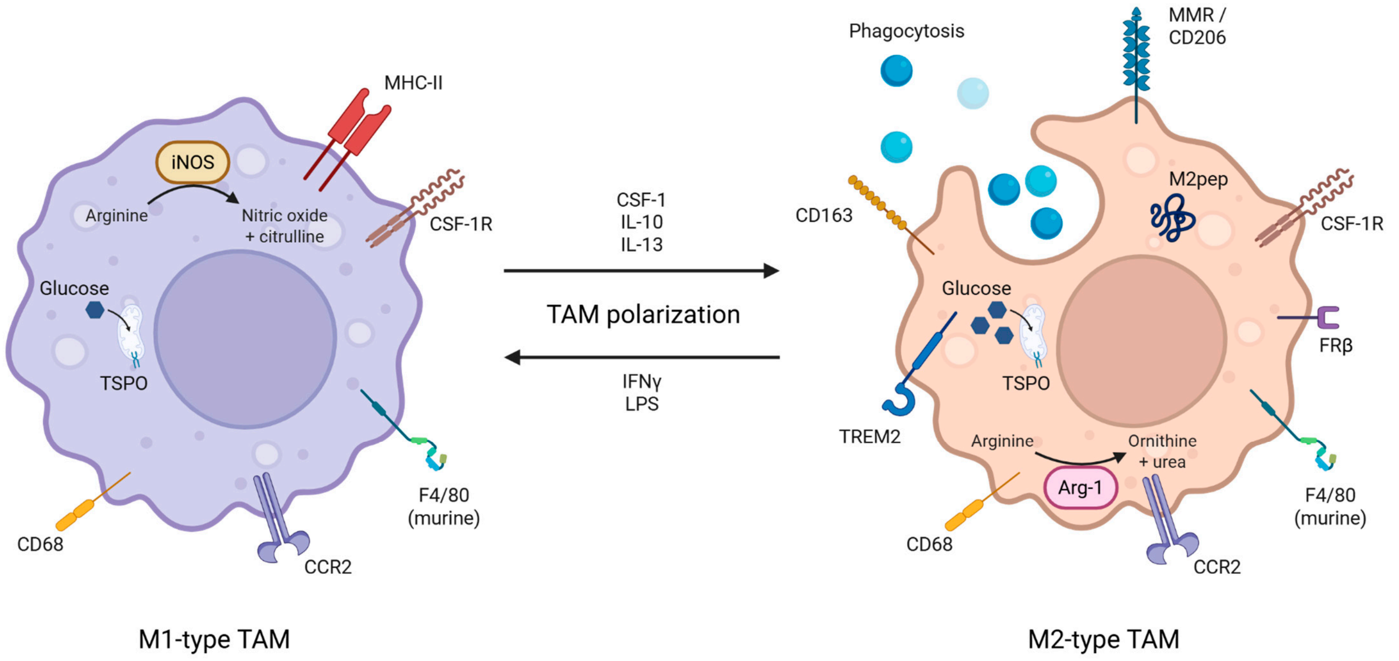

Figure S1. Overview of cell surface and intracellular biomarkers used for radionuclide targeting of TAMs. Arg-1, arginase-1; CCR2, C-C chemokine receptor 2; CSF-1, colony-stimulating factor 1; CSF-1R, colony-stimulating factor 1 receptor; FRβ, folate receptor beta; IFNγ, interferon-gamma; IL, interleukin; iNOS, inducible nitric oxide synthase; LPS, lipopolysaccharide; M2pep, M2 macrophage-targeting peptide; MMR, macrophage mannose receptor; TAM, tumor-associated macrophage; TREM2, triggering receptor expressed on myeloid cells 2; TSPO, translocator protein.

 https://doi.org/10.3390/jcm14145075

Journal of Clinical Medicine
MDPI

2. Supplementary tables

Table S1. Cell surface biomarkers of TAMs

Target	TAM type	Imaging modality	Imaging agent	Study type	Model / Case details	Key findings
**F4/80 receptor**	M1 / M2	SPECT	^{111}In-anti-F4/80-A3-1[10]	Preclinical	MDA-MB-231 xenograft mice	Specifically binds F4/80^{+} macrophages with significant uptake in spleen and tumors. Liposomal clodronate pretreatment reduces liver/spleen uptake.
**CSF-1R**	M1 / M2	PET	^{89}Zr-DFO-CSF1R mAb[12]	Preclinical	KEP Breast cancer mouse model	Depletes TAMs, high uptake in lymphoid tissues.
**CCR2**	M1 / M2	PET	^{64}Cu-DOTA-ECL1i[14]	Preclinical	Spontaneous PDAC GEM models	CCR2-targeted Cu@CuO-ECL1i nanoparticles enable imaging and gemcitabine delivery.
**MMR (CD206)**	M2	SPECT	^{99m}Tc-anti-MMR nanobody[17]	Preclinical	TS/A murine breast cancer / 3LL-R lung cancer models	Targets MMR+ stromal cells with high specificity using nanobodies.
		SPECT	^{125}I-αCD206 and Dye-αCD206[18]	Preclinical	4T1 murine breast cancer in BALB/c normal mice	^{125}I-αCD206 and Dye-αCD206 effectively enable selective imaging of M2-type tumor-associated macrophages by targeting the CD206 receptor.
		PET	^{18}F-FB-anti-MMR 3.49 sdAb[19]	Preclinical	Wild-type, MMR$^{-/-}$, and CCR2$^{-/-}$ mice	High affinity (Kd:1.8-12nM), rapid renal clearance, specific for M2-type TAMs.
		PET	^{68}Ga-NOTA-anti-MMR[20]	Clinical (Phase I)	7 patients with solid tumors (e.g., breast, lung cancer)	Safe with rapid blood clearance. Higher uptake in progressive disease.
		PET	^{68}Ga-NOTA-anti-MMR[21]	Preclinical	Wild-type and MMR-deficient 3LL-R tumor mice	Rapid renal clearance, Safe for Phase I trials.

Journal of
Clinical Medicine

Table S1. Cell surface biomarkers of TAMs (continued)

**TREM2**	M2	PET	^{68}Ga-NOTA-COG1410[27]	Preclinical	Digestive tumor mouse models	Targets TREM2^{+} TAMs, distinguishes tumors from inflammatory regions.
**M2pep**	M2	PET	^{68}Ga-DOTA-M2pep	Preclinical	B16F10 melanoma model	Rapid renal clearance; tumor-to-background ratio>5.

CCR2, C-C chemokine receptor 2; CSF-1R, colony-stimulating factor 1 receptor; MMR, macrophage mannose receptor; M2pep, M2 macrophage-targeting peptide; PDAC, pancreatic ductal adenocarcinoma; PET, positron emission tomography; SPECT, single-photon emission computed tomography; TAM, tumor-associated macrophage; TREM2, Triggering receptor expressed on myeloid cells 2

Journal of Clinical Medicine

MDPI

Table S2. Other TAM-related biomarkers

Target	TAM type	Imaging modality	Imaging agent	Study type	Model / Case details	Key findings
**TSPO**	M1 / M2 / tumor cell	PET	^{18}F-DPA-714[31]	Clinical (Pilot)	13 patients with TNBC	Identifies M2-polarized TAM-rich tumors; TSPO polymorphism affects uptake.
		PET	^{11}C-PBR28[32]	Preclinical	KRAS/p53-mutant PDAC mouse model	Tracks TAM distribution; reduced uptake in CD11b-deficient mice.
**Glucose metabolism**	M1, M2, tumor cell	PET	^{18}F-FDG[34]	clinical	Lung cancer patients	TAM density positively correlates with 18F-FDG uptake; suggests TAMs promote tumor glycolysis and hypoxia.
**HDL**	M2	PET	^{89}Zr-PL-HDL & ^{89}Zr-AI-HDL[37]	Preclinical	Orthotopic breast cancer models	TAM-selective uptake (higher than tumor cells).
**Macrin**	M2	PET	^{64}Cu-Macrin[38]	Preclinical	Lung and breast cancer models	Quantifies TAM heterogeneity; monitors therapy-induced TAM changes.
**Liposome**	M2	PET	^{64}Cu-MAN-LIPs[39]	Preclinical	Lung adenocarcinoma mouse model	Targets TAMs in tumors, potential for drug delivery.

HDL, high-density lipoprotein; PDAC, pancreatic ductal adenocarcinoma; PET, positron emission tomography; TAM, tumor-associated macrophage; TNBC, triple-negative breast cancer; TSPO, Translocator protein
